# Supplementary material for: The generation of HepG2 transmitochondrial cybrids to reveal the role of mitochondrial genotype in idiosyncratic drug-induced liver injury
Source: eLife. 2023 Jun 6;12:e78187. doi: 10.7554/eLife.78187 (PMC10270688; doi:10.7554/eLife.78187)
Supplement: Figure 9—figure supplement 2—source data 1. [file elife-78187-fig9-figsupp2-data1.pptx]

## Slide 1
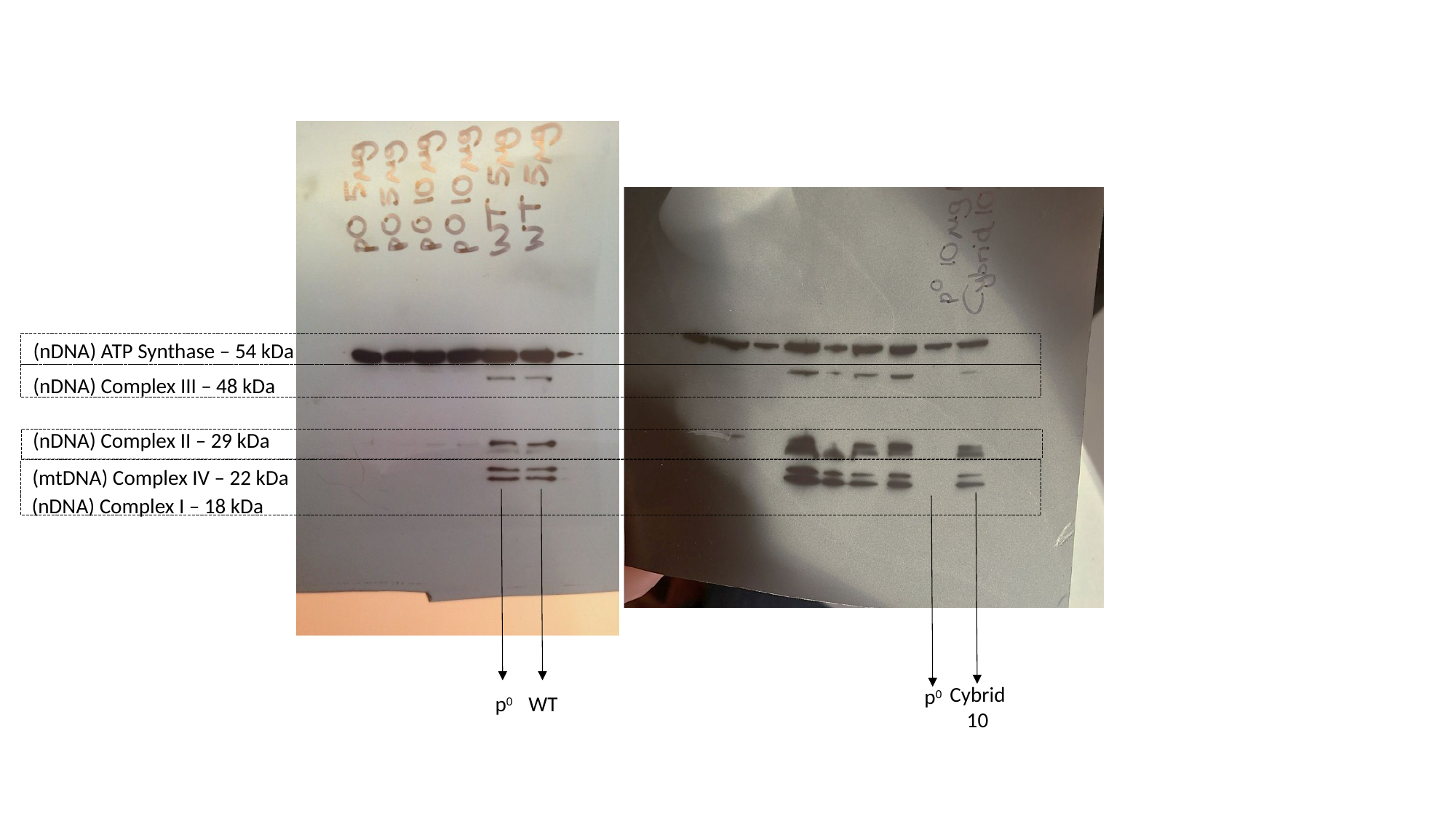

(nDNA) ATP Synthase – 54 kDa
(nDNA) Complex III – 48 kDa
(nDNA) Complex II – 29 kDa
(mtDNA) Complex IV – 22 kDa
(nDNA) Complex I – 18 kDa
Cybrid 10
p0
p0
WT
