# Supplementary figures and images for: The generation of HepG2 transmitochondrial cybrids to reveal the role of mitochondrial genotype in idiosyncratic drug-induced liver injury
Source: eLife. 2023 Jun 6;12:e78187. doi: 10.7554/eLife.78187 (PMC10270688; doi:10.7554/eLife.78187)

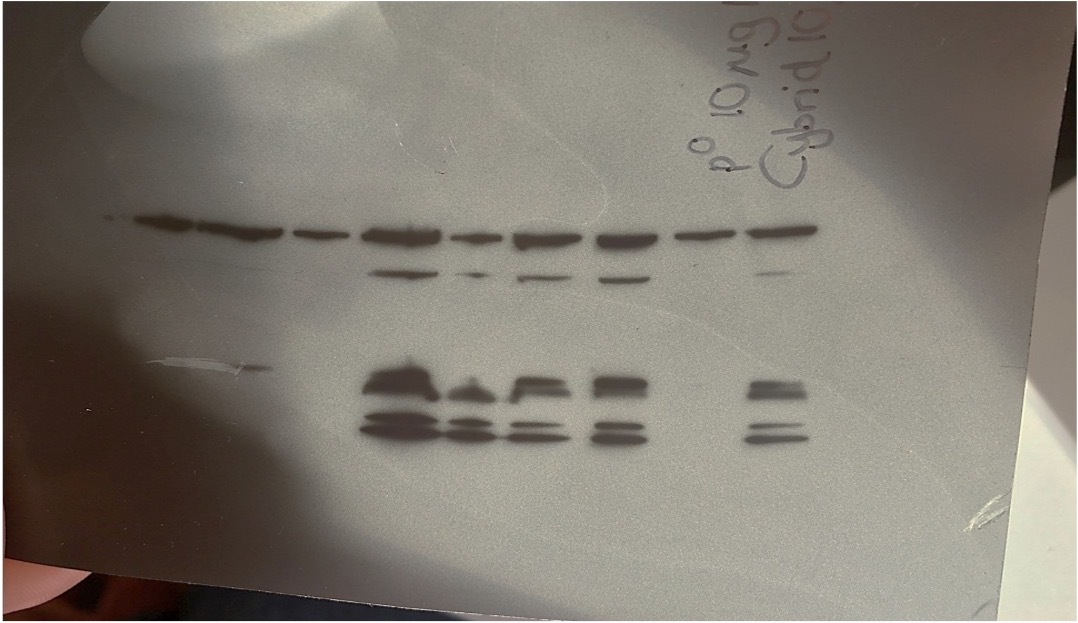

Supplement: Figure 9—figure supplement 2—source data 2. [file elife-78187-fig9-figsupp2-data2.zip › Figs3 blot images/fig s3 blot (p0 and cybrid).jpg]

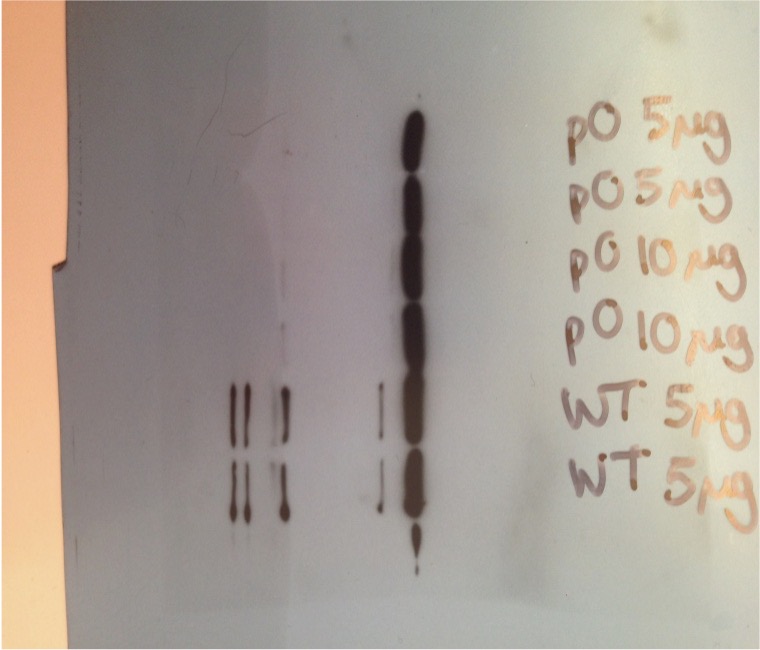

Supplement: Figure 9—figure supplement 2—source data 2. [file elife-78187-fig9-figsupp2-data2.zip › Figs3 blot images/figs3 blot (p0 and WT).jpg]
